# Efficacy and safety of mavrilimumab in giant cell arteritis: a phase 2, randomised, double-blind, placebo-controlled trial

**DOI:** 10.1136/annrheumdis-2021-221865

**Published:** 2022-03-09

**Authors:** Maria C Cid, Sebastian H Unizony, Daniel Blockmans, Elisabeth Brouwer, Lorenzo Dagna, Bhaskar Dasgupta, Bernhard Hellmich, Eamonn Molloy, Carlo Salvarani, Bruce C Trapnell, Kenneth J Warrington, Ian Wicks, Manoj Samant, Teresa Zhou, Lara Pupim, John F Paolini, the KPL-301-C001 Investigators

**Affiliations:** 1 Department of Autoimmune Diseases, Hospital Clinic de Barcelona. University of Barcelona. Institut d'Investigacions Biomèdiques August Pi i Sunyer, Barcelona, Spain; 2 Vasculitis and Glomerulonephritis Center, Division of Rheumatology, Allergy and Immunology, Massachusetts General Hospital, Boston, Massachusetts, USA; 3 Clinical department of General Internal Medicine Department, Research Department of Microbiology and Immunology, Laboratory of Clinical Infectious and Inflammatory Disorders, Katholieke Universiteit Leuven Universitaire Ziekenhuizen Leuven, Leuven, Belgium; 4 Rheumatology and Clinical Immunology, Universitair Medisch Centrum Groningen afdeling Reumatologie & Klinische Immunologie, Groningen, The Netherlands; 5 Vita-Salute San Raffaele University, Milano, Italy; 6 Unit of Immunology, Rheumatology, Allergy and Rare Diseases (UnIRAR), IRCCS San Raffaele Scientific Institute, Milano, Italy; 7 Rheumatology, Mid & South Essex University Hospitals NHS Foundation Trust, Southend University Hospital, Basildon, UK; 8 Klinik für Innere Medizin, Rheumatolgie und Immunologie, Medius KLINIKEN gemeinnutzige GmbH, Kirchheim unter Teck, Germany; 9 Bone and Joint Unit, Saint Vincent's University Hospital, Dublin, Ireland; 10 Unit of Rheumatology, Azienda USL - IRCCS di Reggio Emilia, Reggio Emilia, Italy; 11 Department of Surgery, Medicine, Dentistry and Morphological Sciences with Interest in Transplant, Oncology and Regenerative Medicine, Universita degli Studi di Modena e Reggio Emilia, Modena, Italy; 12 Translational Pulmonary Science Center, Cincinnati Children’s Hospital, Cincinnati, Ohio, USA; 13 Rheumatology, Mayo Clinic, Rochester, Minnesota, USA; 14 Walter and Eliza Hall Institute of Medical Research, Melbourne, Victoria, Australia; 15 Rheumatology Unit, Royal Melbourne Hospital, Parkville, Victoria, Australia; 16 Kiniksa Pharmaceuticals Corp, Lexington, Massachusetts, USA

**Keywords:** giant cell arteritis, glucocorticoids, inflammation, systemic vasculitis

## Abstract

**Objectives:**

Granulocyte-macrophage colony-stimulating factor (GM-CSF) is implicated in pathogenesis of giant cell arteritis. We evaluated the efficacy of the GM-CSF receptor antagonist mavrilimumab in maintaining disease remission.

**Methods:**

This phase 2, double-blind, placebo-controlled trial enrolled patients with biopsy-confirmed or imaging-confirmed giant cell arteritis in 50 centres (North America, Europe, Australia). Active disease within 6 weeks of baseline was required for inclusion. Patients in glucocorticoid-induced remission were randomly assigned (3:2 ratio) to mavrilimumab 150 mg or placebo injected subcutaneously every 2 weeks. Both groups received a 26-week prednisone taper. The primary outcome was time to adjudicated flare by week 26. A prespecified secondary efficacy outcome was sustained remission at week 26 by Kaplan-Meier estimation. Safety was also assessed.

**Results:**

Of 42 mavrilimumab recipients, flare occurred in 19% (n=8). Of 28 placebo recipients, flare occurred in 46% (n=13). Median time to flare (primary outcome) was 25.1 weeks in the placebo group, but the median was not reached in the mavrilimumab group (HR 0.38; 95% CI 0.15 to 0.92; p=0.026). Sustained remission at week 26 was 83% for mavrilimumab and 50% for placebo recipients (p=0.0038). Adverse events occurred in 78.6% (n=33) of mavrilimumab and 89.3% (n=25) of placebo recipients. No deaths or vision loss occurred in either group.

**Conclusions:**

Mavrilimumab plus 26 weeks of prednisone was superior to placebo plus 26 weeks of prednisone for time to flare by week 26 and sustained remission in patients with giant cell arteritis. Longer treatment is needed to determine response durability and quantify the glucocorticoid-sparing potential of mavrilimumab.

**Trial registration number:**

ClinicalTrials.gov number: NCT03827018, Europe (EUdraCT number: 2018-001003-36), and Australia (CT-2018-CTN-01 865-1).

**Key messages**

**What is already known about this subject?**

- Currently available treatments for giant cell arteritis have important limitations. Most patients with giant cell arteritis treated with glucocorticoids alone experience disease relapse and/or develop glucocorticoid-related toxicity, and a significant proportion of patients treated with tocilizumab cannot achieve sustained remission or must discontinue this medication due to adverse events.
- Translational research has implicated granulocyte-macrophage colony-stimulating factor (GM-CSF) in the pathogenesis of giant cell arteritis, with studies showing upregulation of the GM-CSF and TH1/TH17 pathways in temporal arteries of patients with giant cell arteritis and amelioration of the abnormal immune response (eg, inflammatory cell infiltration and expression of interferon-γ and interleukin-6) on GM-CSF signalling blockade with mavrilimumab.

**What does this study add?**

- This study demonstrated that mavrilimumab in combination with a 26-week prednisone taper was superior to placebo with a 26-week prednisone taper in reducing the risk of flare and maintaining sustained remission and was well tolerated.

**How might this impact on clinical practice or future developments?**

- The study findings support the hypothesis that GM-CSF signalling activates important pathways in the pathogenesis of giant cell arteritis, and that inhibition of these pathways by GM-CSF receptor blockade with mavrilimumab might maintain remission of the disease.
- These phase 2 results are encouraging for the further development of mavrilimumab as a potential treatment for giant cell arteritis.

## Introduction

Giant cell arteritis (GCA) is the most prevalent form of systemic vasculitis in adults.^1^ The disease is driven by CD4^+^ T-cells (T helper (T_h_) 1 and 17 cells) and macrophages that infiltrate large-sized and medium-sized arteries.^2 3^ Clinical manifestations include headaches, jaw claudication, ocular ischaemia, polymyalgia rheumatica and constitutional symptoms.^1 4^ Possible complications include blindness and aortic aneurysms.^1^ Most patients with active GCA exhibit elevated acute-phase reactants, including erythrocyte sedimentation rate (ESR) and serum C reactive protein (CRP) levels,^5^ that, along with serial assessment of clinical manifestations, are useful in monitoring disease activity.^1^

Therapeutic options that safely maintain disease remission in patients with GCA are limited.^6^ When treated with glucocorticoids alone, approximately 34%–75% of patients experience disease flare on dose reduction or drug discontinuation.^4 7 8^ Moreover, the prolonged treatment with glucocorticoids required to control the disease, usually more than 12–18 months, causes significant glucocorticoid-related toxicity in the majority of patients.^9 10^ Tocilizumab in combination with ≥6 months of glucocorticoids has demonstrated efficacy in maintaining disease remission and sparing the use of glucocorticoids and is the only approved adjuvant treatment for GCA patients. Unfortunately, 24%–30% of patients receiving tocilizumab flare within 1 year, and approximately 5%–8% of them must discontinue treatment because of side effects.^11 12^ Also, given the direct suppression of hepatic acute-phase reactant synthesis, tocilizumab renders ESR and CRP unreliable for monitoring of disease activity and potential intercurrent infectious complications.^13 14^ Other medications which have been tried for GCA, such as methotrexate and abatacept, have demonstrated modest benefits at best or need confirmation.^15–17^ Therefore, novel treatments that safely maintain remission of GCA while allowing for acute-phase reactant monitoring are needed.

Granulocyte-macrophage colony-stimulating factor (GM-CSF) is a multifunctional cytokine that modulates the biology of dendritic cells, CD4^+^ T-cells and macrophages.^18^ Preclinical research has implicated GM-CSF in the pathogenesis of GCA.^19–22^ GM-CSF, its receptor, and downstream signalling molecules are expressed by immune and endothelial cells in temporal arteries from patients.^19–22^ Furthermore, GM-CSF receptor blockade in cultured temporal arteries resulted in decreased expression of dendritic cell, T-cell, and macrophage markers along with downregulation in transcription of genes associated with the T_h_1 and T_h_17 immune responses (eg, interferon-γ and interleukin-6).^20 22^ In a mouse model of vascular inflammation, GM-CSF inhibition was associated with reduced arterial inflammation and remodelling.^23^ Mavrilimumab, an immunoglobulin G4 monoclonal antibody with demonstrated efficacy in phase 2 studies of rheumatoid arthritis,^24 25^ blocks GM-CSF signalling by binding to the alpha chain of the receptor.

We conducted a proof-of-concept, randomised, double-blind, placebo-controlled trial to investigate whether mavrilimumab reduced the risk of GCA flare compared with placebo, during a 26-week glucocorticoid taper.

## Methods

### Study design

This randomised, double-blind, placebo-controlled phase 2 trial was conducted in 50 centres across 15 countries in North America, Europe, and Australia.

### Patients

Patients age 50–85 years with new-onset (diagnosis ≤6 weeks before baseline) or relapsing/refractory (diagnosis >6 weeks before baseline) GCA and active disease within 6 weeks of randomisation were eligible. Active disease was defined as the presence of one or more clinical manifestations, including cranial (eg, headache, scalp or temporal artery tenderness, new/worsening ischaemia-related visual impairment or jaw claudication) or extracranial (eg, new/worsening extremity claudication or polymyalgia rheumatica) signs or symptoms, plus Westergren ESR ≥30 mm per hour or a CRP level ≥1 mg per decilitre. Isolated ESR or CRP elevation was not considered active disease for patient enrolment. GCA diagnosis was confirmed based on a temporal artery biopsy showing GCA features or by findings indicative of vasculitis on temporal artery ultrasonography or large-vessel imaging including magnetic resonance angiography, computed tomography (CT) angiography or positron emission tomography/CT. Complete eligibility criteria are detailed in online supplemental methods.

10.1136/annrheumdis-2021-221865.supp1 Supplementary data

### Procedures

**Figure 1:**
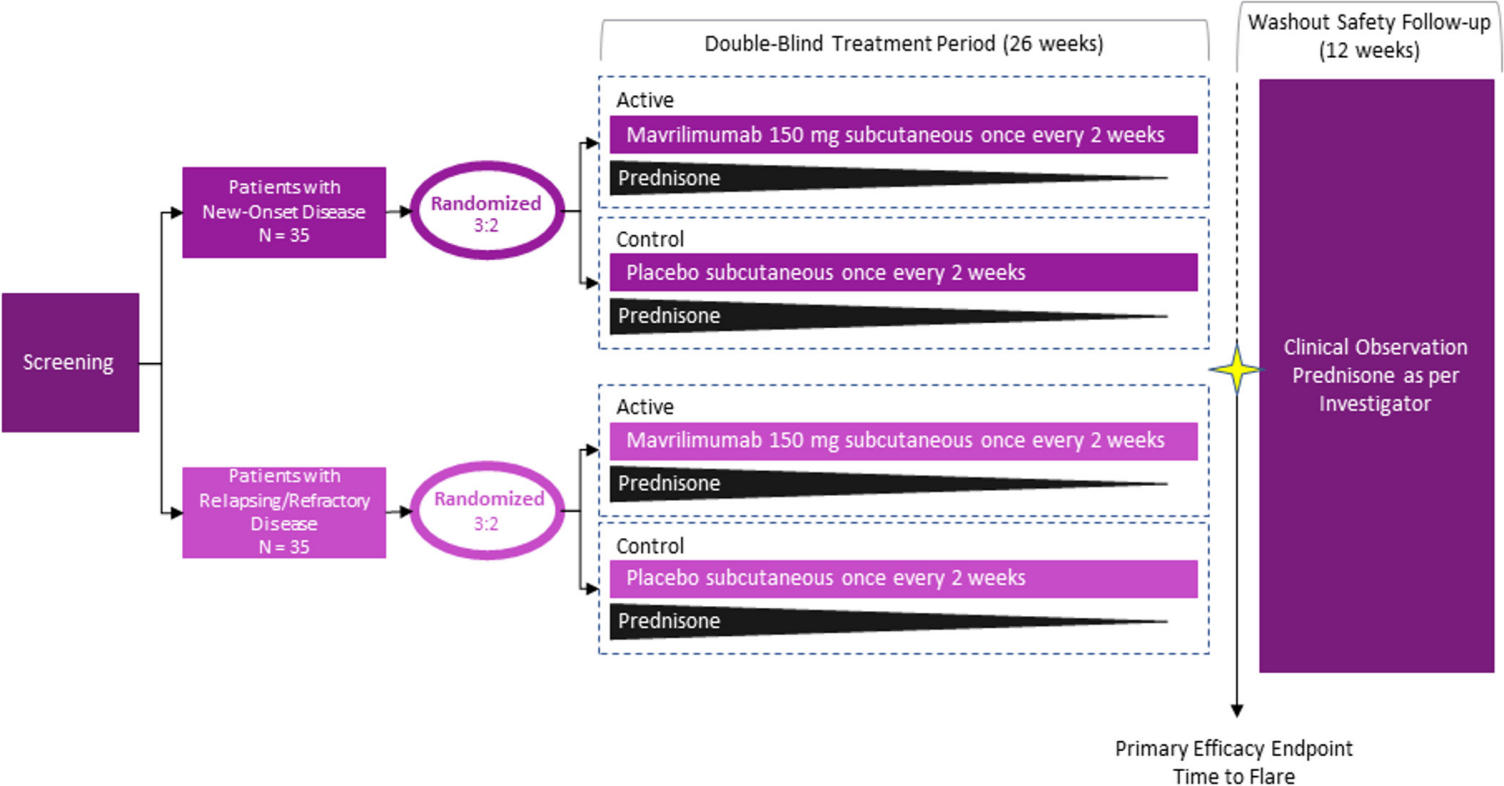
Trial design. Patients were randomised in a 3:2 ratio to mavrilimumab or placebo using disease type (new onset or relapsing/refractory) as a stratification factor. Prednisone was tapered over the 26-week study as specified in the protocol.

Following a screening period (≤6 weeks), eligible patients were randomly assigned in a 3:2 ratio to mavrilimumab 150 mg or placebo subcutaneously every other week with a 26-week prednisone taper and entered a double-blind, placebo-controlled treatment period (26 weeks), which was followed by a safety follow-up period (12 weeks) (figure 1). Given that in prior 1-year trials with 26-week steroid tapers^11 16^ the majority of disease flares occurred within the first 6 months, we limited the treatment period of this proof-of-concept trial to 26 weeks to expedite the generation of efficacy results. Randomisation was stratified by disease type (new onset or relapsing/refractory) at baseline. At baseline, patients were required to be in glucocorticoid-induced remission and on an oral prednisone dose between 20 and 60 mg daily. Remission at baseline was defined as the absence of disease signs and symptoms and ESR<20 mm per hour or serum CRP concentration <1 mg per decilitre. From baseline, the prednisone dose was tapered weekly in both groups as stipulated by the protocol. Additional details can be found in online supplemental file.

### Outcomes

#### Efficacy

Patients were assessed at planned study visits and during unscheduled visits to determine disease remission status and whether the protocol prednisone taper could continue. It was recommended that the investigator evaluate signs and symptoms of GCA before reviewing laboratory or imaging results to minimise potential bias. ESR and/or CRP levels were measured locally. Patients requiring treatment for flare during the double-blind period discontinued study drug and received standard treatment, including glucocorticoids, as per the investigators’ clinical judgement. After the 26-week treatment period, patients discontinued study drug and transitioned to standard of care, which could include glucocorticoids, during a 12-week washout period. Patients were closely monitored for safety and flare through week 38.

The primary efficacy end point was time to first GCA flare by week 26. Flare was centrally adjudicated by an independent, blinded clinical end point adjudication committee and defined as elevation of ESR (≥30 mm/hour) and/or CRP (≥1 mg/dL) along with either the presence of unequivocal cranial or extracranial signs or symptoms or the occurrence of new or worsening imaging abnormalities suggestive of active vasculitis. ESR or CRP elevation was not considered disease flare in the absence of signs, symptoms or imaging abnormalities suggesting disease activity. Further details of flare adjudication are included in online supplemental methods.

A key prespecified secondary efficacy end point was sustained remission rate at week 26 using Kaplan-Meier estimation, which was defined as the absence of flare from randomisation through week 26. Time to flare and sustained remission by week 26 were also assessed in the subgroups of patients with new-onset and relapsing/refractory disease at baseline. Cumulative prednisone dose by treatment arm was assessed. The proportion of patients with elevated ESR or CRP but without giant-cell arteritis flare was assessed in a post hoc analysis. Additional secondary end points and their hierarchy are described in online supplemental methods.

#### Safety

Safety was assessed through week 38 for all patients who received at least one mavrilimumab or placebo dose. Incidence, severity, and relationship of adverse events to study drug were summarised by treatment group. A data-monitoring committee periodically reviewed all safety data during the trial. Patients underwent serial pulmonary function testing and completed the modified Borg Dyspnoea Scale^26^ at regular intervals. An independent committee adjudicated pulmonary adverse events of special interest including the potential occurrence of pulmonary alveolar proteinosis.^27^

### Statistical analyses

A sample size of approximately 70 patients was determined based on an assumption, consistent with literature data, that 50% of placebo recipients and 15% of mavrilimumab recipients would flare by week 26, with a median time to flare of approximately 26 weeks in placebo group and 111 weeks in mavrilimumab group, corresponding with an HR of approximately 0.234. Using a time-to-flare model and a 3:2 randomisation ratio, we calculated that 20 flares would give the trial 87% power to detect a significant difference between treatment groups with a two-sided alpha level of 0.05. The analysis of the new onset and relapsing/refractory subgroups, while prespecified, was not powered for significance. The efficacy end point analysis was performed in the modified intention-to-treat population, which included all randomised patients who had received at least one dose of study treatment and had at least one assessment in the double-blind treatment period. The primary end point and other time-to-event end points were summarised with percentiles and 95% CIs using the Kaplan-Meier method. Patients without a flare were censored at the last assessment by week 26 or by end of treatment visit, in case of early treatment discontinuation, for calculation of the time to flare. A log-rank test stratified by disease type (new onset vs relapsing/refractory) at baseline was used to compare mavrilimumab with placebo. The number and percentage of patients who had a flare during the 26-week double-blind period were summarised for each treatment group. A Cox proportional hazards model was used to calculate hazard ratios and 95% CIs. Sustained remission at week 26 was derived by Kaplan-Meier curve analysis.

All secondary outcomes based on proportions were assessed using the Cochran-Mantel-Haenszel test.

A gatekeeping multiplicity-adjustment procedure in combination with the Hochberg method was applied for prespecified stepwise testing of the primary end point and the secondary end points. If the two-sided p value for an end point (highest in hierarchy) was no more than 0.05, the next prespecified end point in the hierarchy would be tested at the same alpha level. Details of hierarchy are provided in online supplemental methods.

## Results

### Patients

**Figure 2:**
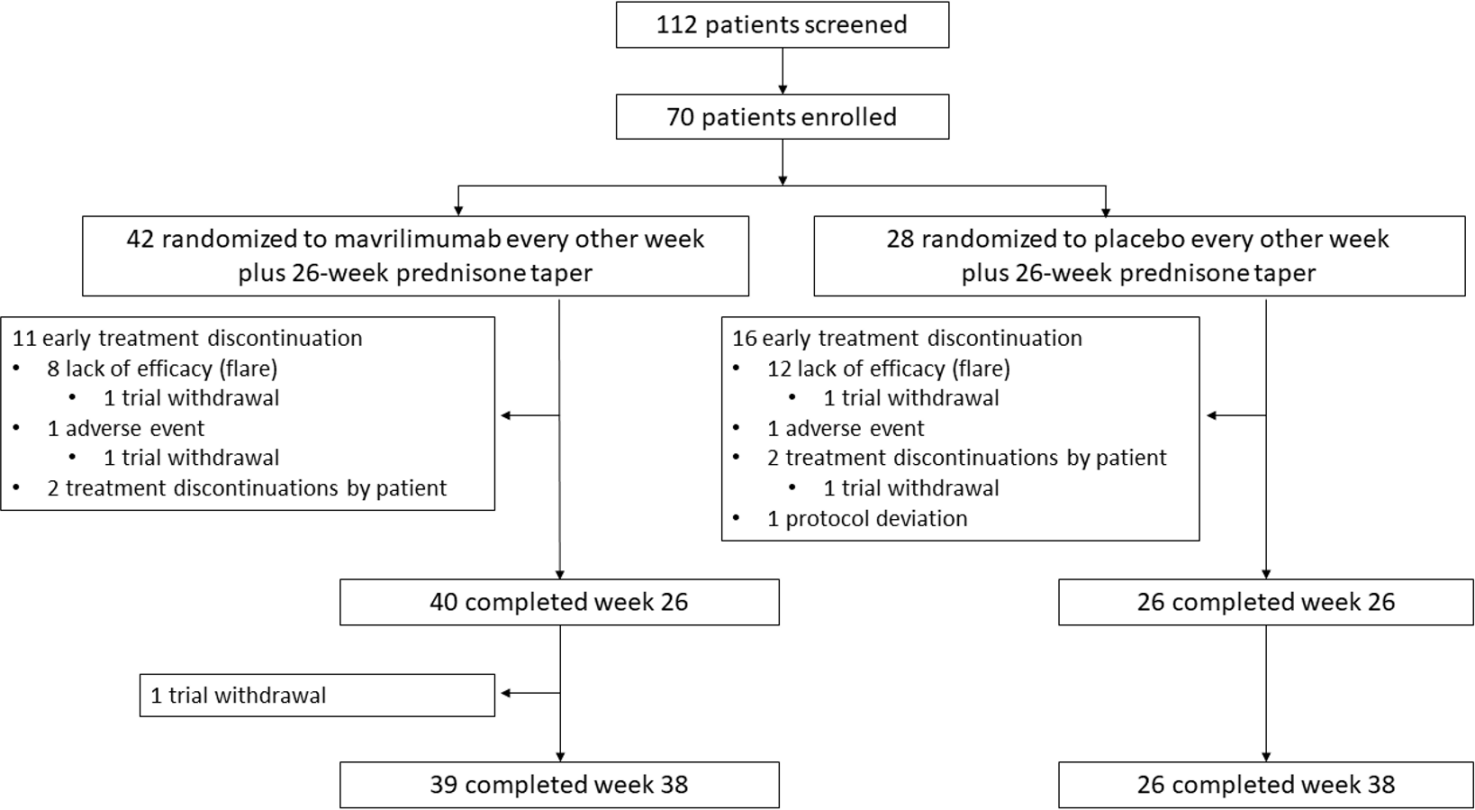
Trial profile. Not all patients who discontinued treatment withdrew from the trial; two patients receiving mavrilimumab and two patients receiving placebo withdrew before week 26, and one patient receiving mavrilimumab withdrew between week 26 and week 38.

Of 112 patients assessed for eligibility, 70 were enrolled in the trial between 20 September 2018 and 27 January 2020. Figure 1 shows the clinical trial schema. A total of 42 patients were randomly assigned to mavrilimumab and 28 to placebo. The demographic and baseline characteristics of the treatment groups are displayed in table 1. GCA diagnosis was confirmed by biopsy in 31 (44%) patients and by imaging in 51 (73%) patients. A total of 66 patients completed the 26-week study period (figure 2).

**Table 1:** Baseline characteristics of the intention-to-treat population†

	Mavrilimumab‡ (n=42)	Placebo (n=28)
Age (years)	69.7 (7.0)	69.7 (8.3)
Sex		
Male	10 (24%)	10 (36%)
Female	32 (76%)	18 (64%)
Race		
White	40 (95%)	28 (100%)
Other	2 (5%)	0
Hispanic or Latino ethnicity	1 (2%)	2 (7%)
Weight (kg)	70.9 (18.7)	71.1 (12.0)
Body mass index (kg/m^2^)	26.2 (6.8)	26.1 (3.6)
Prior treatment		
Glucocorticoids	42 (100%)	27 (96%)
Methotrexate	0	1 (4%)
Diagnostic confirmation		
By positive temporal artery biopsy	22 (52%)	9 (32%)
By positive imaging	29 (69%)	22 (79%)
Time since diagnosis (months)	7.9 (15.4)	9.8 (21.8)
Giant-cell arteritis		
New onset*	24 (57%)	11 (39%)
Relapsing/refractory*	18 (43%)	17 (61%)
Giant-cell arteritis type		
Cranial signs or symptoms	32 (76%)	21 (75%)
Extracranial signs or symptoms	9 (21%)	6 (21%)
C reactive protein level (study eligibility value) (mg/dL)	4.7 (4.7)	3.6 (3.2)
Erythrocyte sedimentation rate (study eligibility value) (mm/hour)	57.0 (24.6)	55.1 (30.2)
Prednisone starting dose		
≤30 mg	16 (38.1)	14 (50.0)
>30 mg	26 (61.9)	14 (50.0)

Data are n (%) or mean (SD).

*Seven patients were misstratified due to investigator error (new onset vs relapsing/refractory misclassification) at study entry. For the efficacy analysis, these patients were included in the appropriate protocol-defined subgroups, leading to a proportion of 57% of patients with new-onset disease in the mavrilimumab group (43% relapsing/refractory) and 39% of patients with new-onset disease in the placebo group (61% relapsing/refractory).

†Baseline is last assessment within 3 days before the first dose of mavrilimumab or placebo.

‡150 mg subcutaneously every 2 weeks.

### Primary and key secondary efficacy outcomes

**Figure 3:**
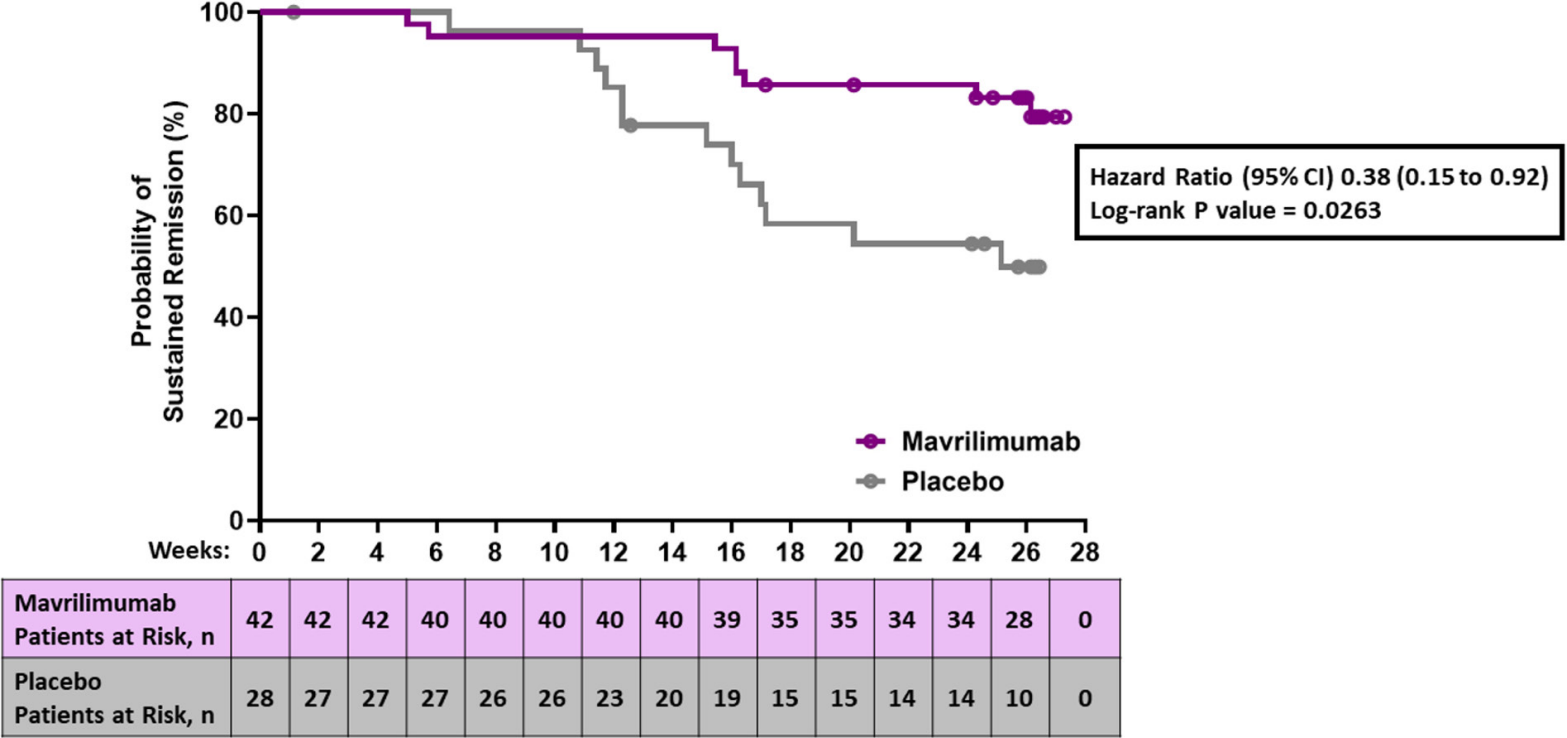
Time to first flare of giant-cell arteritis in all patients. At baseline, patients had to be in remission (defined as the absence of giant-cell arteritis signs and symptoms and erythrocyte sedimentation rate <20 mm/hour or C reactive protein level <1 mg/dL) and receiving an oral prednisone dose between 20 mg and 60 mg daily. Patients who discontinued treatment for reasons other than flare were censored for the calculation of time to flare. The median time to flare could not be calculated for patients receiving mavrilimumab because fewer than 50% of patients experienced a flare during the 26 weeks study period.

**Figure 4:**
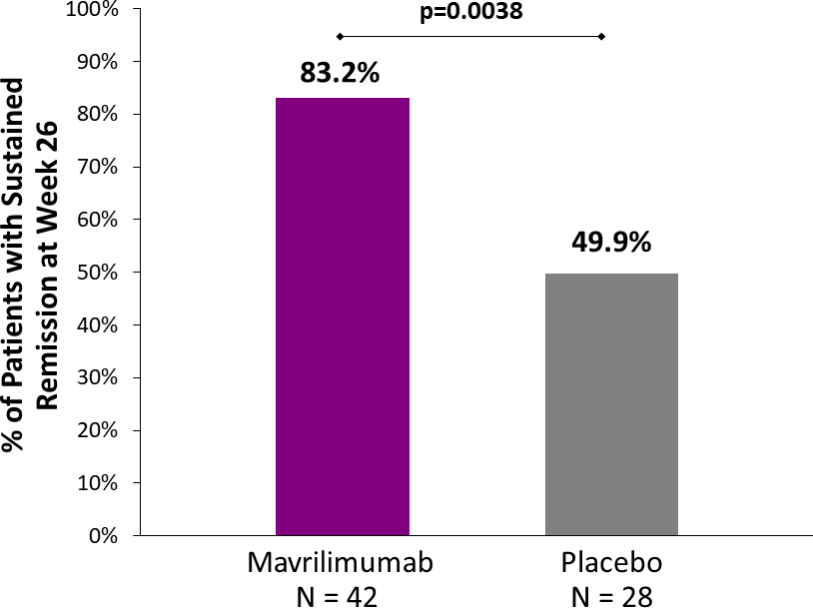
Sustained remission rate of giant-cell arteritis in all patients at week 26. The difference in sustained remission at week 26 (key secondary endpoint) was statistically significant (33.3 percentage points; p=0.0038). Sustained remission was defined as the absence of flare from randomisation through week 26. Sustained remission rate was derived by Kaplan-Meier curve analysis.

During the 26-week placebo-controlled period, 21 patients developed an adjudicated flare: eight (19%) mavrilimumab recipients and 13 (46.4%) placebo recipients. GCA signs or symptoms were present in 20 of the 21 patients with flare; in the one other patient, flare was determined based on presence of active vasculitis on ultrasound imaging. Median time to flare (primary end point) in placebo recipients was 25.1 weeks (95% CI 16.0 to not estimable (NE)). The median time to flare among mavrilimumab recipients was not reached within the 26-week follow-up period. Mavrilimumab reduced the risk of flare vs placebo (HR, 0.38; 95% CI 0.15 to 0.92; p=0.026) (figure 3). Sustained remission at week 26 (key secondary end point) was reached in 83.2% of mavrilimumab recipients and 49.9% of placebo recipients (33.3 percentage points difference; p=0.0038) (figure 4, table 2). Detailed flare information is provided in online supplemental table S1.

**Table 2:** Primary end point and key secondary end points

End point	Mavrilimumab**	Placebo	HR or difference	P value*
All study patients†	(N=42)	(N=28)	−	−
Patients with flare	8 (19.0%)	13 (46.4%)	−	−
Time to flare (primary end point)—week	NE (NE, NE)	25.1 (16.0 to NE)	0.38 (0.15 to 0.92)‡	0.026
Sustained remission§—%	83.2 (67.9 to 91.6)	49.9 (29.6 to 67.3)	33.3 (10.7 to 55.8)¶	0.0038
Patients with new-onset† giant-cell arteritis at baseline	(N=24)	(N=11)	−	−
Patients with flare	3 (12.5%)	4 (36.4%)	−	−
Time to flare—week	NE (NE to NE)	NE (11.7 to NE)	0.29 (0.06 to 1.31)‡	−
Sustained remission§—%	91.3 (69.3 to 97.7)	62.3 (27.7 to 84.0)	28.9 (−2.7 to 60.5)¶	−
Patients with relapsing/refractory† giant-cell arteritis at baseline	(N=18)	(N=17)	−	−
Patients with flare	5 (27.8%)	9 (52.9%)	−	−
Time to flare—week	NE (16.4 to NE)	22.6 (16.0 to NE)	0.43 (0.14 to 1.30)‡	−
Sustained remission§—%	72.2 (45.6 to 87.4)	41.7 (17.4 to 64.5)	30.6 (−2.1 to 63.2)¶	−

Data are n (%) or median (95% CI), except as indicated.

*P values are two sided.

†Modified intention-to-treat (mITT) population.

‡Calculated using a Cox proportional hazards model with treatment as covariate.

§The Kaplan-Meier method was used to estimate event rates. In some cases, results were NE because the event rates were too low.

¶Calculated as the difference in sustained remission between the two groups using normal approximation with placebo as the reference.

**150 mg subcutaneously every 2 weeks.

NE, not estimable.

### New-onset and relapsing/refractory disease

Among the subgroup of patients with new-onset GCA at baseline, flare occurred in 12.5% of mavrilimumab recipients and 36.4% of placebo recipients (HR, 0.29; 95% CI, 0.06 to 1.31) (table 2; online supplemental figure S1A); 91.3% of mavrilimumab recipients and 62.3% of placebo recipients had sustained remission at week 26 (table 2, online supplemental figure S2A). Among the subgroup of patients with relapsing/refractory disease at baseline, flares occurred in 27.8% of mavrilimumab recipients and 52.9% of placebo recipients (HR, 0.43; 95% CI 0.14 to 1.30) (table 2; online supplemental figure S1B); sustained remission at week 26 was observed in 72.2% of mavrilimumab recipients and 41.7% of placebo recipients (table 2, online supplemental figure S2B).

### Cumulative prednisone dose

The mean cumulative prednisone dose by week 26 was 2074 mg in mavrilimumab recipients and 2403 mg in placebo recipients (nominal p=0.067); least-squares mean difference (nominal 95% CI) was –326 mg (–676 mg to 23 mg). Additional secondary end points assessed at week 26 are reported in table 3 and the online supplemental results.

**Table 3:** Other secondary end points

End point	Mavrilimumab* (N=42)	Placebo (N=28)	P value
Time to elevated erythrocyte sedimentation rate by week 26,† median (95% CI) weeks‡	26.1 (16.1, NE)	12.1 (8.1, 16.6)	0.028§
Time to elevated C reactive protein level by week 26,¶ median (95% CI) weeks‡	NE (8.1, NE)	12.3 (3.3, 24.1)	0.038§
Time to signs and symptoms of giant-cell arteritis or new or worsening vasculitis by imaging by week 26, median (95% CI) weeks‡	NE (NE, NE)	25.1 (15.1, NE)	0.065§
Cumulative prednisone dose at week 26, mean (SD) mg	2074 (708)	2403 (1014)	0.067**
Percentage of patients completing glucocorticoid taper†† and with normal erythrocyte sedimentation rate by week 26	19 (45.2%)	4 (14.3%)	0.020**
Percentage of patients completing glucocorticoid taper†† and with normal C reactive protein level by week 26	10 (23.8%)	4 (14.3%)	0.55**
Percentage of patients completing glucocorticoid taper†† and with no signs or symptoms of giant-cell arteritis by week 26	30 (71.4%)	9 (32.1%)	0.0031**
Cumulative prednisone dose at week 38‡‡, mean (SD) mg	2465 (1107)	2845 (1320)	0.16**

Data are n (%) except as indicated.

*150 mg subcutaneously every 2 weeks.

†Elevated erythrocyte sedimentation rate is defined as the first rate greater than or equal to 30 mm/hour; patients with an elevated rate within 3 days of the first dose of study drug were excluded from the analysis.

‡Kaplan-Meier method.

§Log-rank test stratified by randomisation strata.

¶Elevated C reactive protein level is defined as the first level greater than or equal to 1.0 mg/dL; patients with an elevated level within 3 days of the first dose were excluded from the analysis.

**Analysed by Cochran-Mantel-Haenszel test stratified by randomisation strata. Nominal p value.

††Patients were considered to have completed glucocorticoid taper if by week 26 they were receiving 1 mg/day for patients who had a starting dose of 60 mg/day, or 0 mg/day for patients who had a starting dose of less than 60 mg/day.

‡‡After the 26-week treatment period, investigators could manage disease in patients at their discretion, including use of glucocorticoids.

### Acute-phase reactants

Among the 21 patients who had a flare, all had increased ESR or CRP values at the time of flare (by pre-specified flare definition); the median (IQR) CRP level was 1.8 (1.4–6.3) mg per decilitre in mavrilimumab recipients and 1.8 (1.2–2.8) mg per decilitre in placebo recipients. Corresponding ESR values were 40 (33 –73) mm per hour in mavrilimumab recipients and 49 (33–51) mm per hour in placebo recipients (online supplemental table S2). Among 34 mavrilimumab recipients who did not have a flare, 47.1% had at least one elevated ESR (≥30 mm/hour) and 29.4% had at least one elevated CRP (≥1 mg/dL) value through week 26. Among 15 placebo recipients who did not have a flare, 66.7% had at least one elevated ESR and 73.3% had at least one elevated CRP value through week 26 (online supplemental table S2).

### Safety

Adverse events were reported in 78.6% of mavrilimumab recipients and 89.3% of placebo recipients (table 4). Serious adverse events, all unrelated to study drug, were reported in 4.8% of mavrilimumab recipients (one case each of hypertrophic cardiomyopathy and dementia) and 10.7% of placebo recipients (one case each of gastrointestinal haemorrhage, peripheral oedema and pulmonary fibrosis). No adverse event resulted in permanent vision loss or death in either treatment group. Adverse events leading to study drug discontinuation occurred in one patient in each treatment group: dementia in a mavrilimumab recipient and chest pain in a placebo recipient.

**Table 4:** Treatment-emergent adverse events

Adverse events	Mavrilimumab* (N=42)	Placebo (N=28)
Patients with ≥1 adverse event	33 (78.6%)	25 (89.3%)
Serious adverse event	2 (4.8%)	3 (10.7%)
Serious adverse event related to study drug	0	0
Adverse event resulting in death	0	0
Adverse event leading to study drug discontinuation	1 (2.4%)	1 (3.6%)
Adverse events by maximum severity†		
Mild	18 (42.9%)	13 (46.4%)
Moderate	14 (33.3%)	11 (39.3%)
Severe	1 (2.4%)	1 (3.6%)
Most common adverse events‡		
Headache	6 (14.3%)	7 (25.0%)
Nasopharyngitis	5 (11.9%)	3 (10.7%)
Neck pain	4 (9.5%)	2 (7.1%)
Arthralgia	2 (4.8%)	4 (14.3%)
Hypertension	1 (2.4%)	4 (14.3%)
Back pain	3 (7.1%)	3 (10.7%)
Muscle spasms	3 (7.1%)	3 (10.7%)
Upper respiratory tract infection	3 (7.1%)	2 (7.1%)
Constipation	3 (7.1%)	0
Diarrhoea	0	3 (10.7%)
Fall	2 (4.8%)	5 (17.9%)

Data are n (%).

*150 mg subcutaneously every 2 weeks.

†Each patient is represented only with maximum severity.

‡Reported in >2 patients in either treatment group.

The most frequent non-serious adverse events in mavrilimumab recipients were non-specific headache, nasopharyngitis and neck pain. Infections were reported in 42.9% of mavrilimumab recipients and 35.7% of placebo recipients. No serious or severe infections occurred during the trial. Respiratory adverse events were reported in similar proportions in the treatment groups (mavrilimumab, 11.9%; placebo, 10.7%). In mavrilimumab recipients, these included mild cough, mild dyspnoea and mild vasomotor rhinitis. There were no substantive differences between treatment groups in pulmonary function tests, including diffusing capacity of the lung for carbon monoxide, and no cases of pulmonary alveolar proteinosis occurred.

## Discussion

This trial provides the first evidence of the efficacy and safety of mavrilimumab in patients with GCA. Mavrilimumab with a 26-week prednisone taper was superior to placebo with a 26-week prednisone taper in reducing the risk of flare and maintaining sustained remission. Consistent efficacy trends were observed in new-onset and relapsing/refractory disease subgroups, although this analysis was not powered for statistical significance. Mavrilimumab was well tolerated, and the overall incidence of adverse events and serious adverse events was similar between groups.

GCA treatments that safely maintain disease remission are lacking.^6^ The clinical course of patients treated exclusively with glucocorticoids is complicated by high rates of disease flare and increased incidence of glucocorticoid-related toxicity.^4 7 8^ Tocilizumab is the only GCA medication with confirmed, clinically meaningful efficacy in terms of remission maintenance and glucocorticoid-sparing.^11^ However, 24%–30% of patients receiving tocilizumab flare within 1 year, and approximately 5%–8% of them must discontinue treatment because of side effects.^11–13^ In this study, mavrilimumab reduced the risk of flare without adverse events of serious infection or pulmonary alveolar proteinosis,^28^ becoming a promising option for further development in a field in which alternative treatments are a great unmet need.

It is well recognised that the elevation of ESR or serum CRP is not completely sensitive or specific for the diagnosis of GCA flare.^5 13^ However, these acute-phase reactants have been widely used by clinicians as one of several practical elements for monitoring disease activity status in steroid-treated patients. Because tocilizumab reduces IL-6 activity in the liver, it directly inhibits hepatic synthesis of acute-phase reactants and reduces ESR and CRP independently of its immunomodulatory action,^13^ rendering these biomarkers unreliable for monitoring disease activity.^13^ The fact that flares in this trial were associated with increased acute-phase reactants regardless of whether patients were on mavrilimumab or only glucocorticoids suggests that ESR and CRP retained their clinical diagnostic value during GM-CSF blockade.

The safety profile of mavrilimumab was consistent with that observed in larger, long-term studies of patients with rheumatoid arthritis.^25 29^ In this phase 2 trial, mavrilimumab was well tolerated, and most adverse events were mild or moderate. Because GM-CSF plays an important role in lung homeostasis by promoting alveolar macrophage-induced surfactant clearance,^27 28^ respiratory adverse events, including changes in lung function, were assessed by an independent pulmonary evaluation committee. Of note, there were no differences in pulmonary function tests between treatment groups and no cases of pulmonary alveolar proteinosis occurred during the trial.

The design of this phase 2 study incorporated strategic development-phase-specific trade-offs in strengths and limitations as well as guidance provided by regulatory agencies during review of the protocol. On the one hand, informed by the timing of disease flare in other trials,^11 16^ the proposed 26-week placebo-controlled treatment period allowed for expedited generation of proof-of-concept data. The time-to-event variable of time-to-flare was chosen for the primary end point (as opposed to disease remission at a given timepoint) because it would allow for a more comprehensive interpretation of the results by adding the domain of time and the event cadence to the cumulative crude event rates. On the other hand, a period longer than 26 weeks would have been ideal to properly assess long-term remission maintenance and glucocorticoid sparing, important treatment objectives for this chronic, relapsing disease. In this trial, the mean cumulative prednisone dose was lower in mavrilimumab recipients than in placebo recipients, due to higher disease flare and glucocorticoid rescue rates in patients in the placebo group. The difference between groups through week 26, however, did not reach statistical significance, likely because of the late time-to-flare (median 25.1 weeks) in the placebo group relative to the 26-week time point at which the assessment of cumulative prednisone dose ended.

A slight imbalance in the number of patients with new-onset and relapsing/refractory disease between groups could have influenced the results to some extent and may represent a limitation of the study. Although, such possibility seems unlikely in view of prior research demonstrating that duration of disease and the status of newly diagnosed vs relapsing disease do not independently predict treatment failure,^30^ confirmation of these phase 2 results in a larger trial with well-balanced baseline features is required.

Current medications for GCA (eg, glucocorticoids and tocilizumab) target primarily the CD4^+^ T_h_17 immune response, possibly leaving residual CD4^+^ T_h_1 pathway activity, which may explain why a sizeable proportion of patients flare with these treatments. In contrast, GM-CSF blockade with mavrilimumab may address the pathogenic mechanisms of GCA more comprehensively via its demonstrated suppressive effects on macrophages, CD4^+^ T_h_17 cells, and CD4^+^ T_h_1 cells, including downregulation of IFNγ expression.^22 23^ However, further mechanistic research linked to clinical outcomes is needed before firm conclusions can be drawn.

In summary, mavrilimumab given with a 26-week prednisone taper significantly reduced the risk of flare and improved the sustained remission rates compared with placebo with a 26-week prednisone taper in patients with GCA. Mavrilimumab was well tolerated, and no new safety signals emerged in this clinical trial. These results are supportive of further clinical development of mavrilimumab; confirmation of these overall results, precise distinction of efficacy in new-onset and relapsing/refractory disease subgroups, and determination of response durability and glucocorticoid-sparing potential should all be addressable in a larger pivotal clinical trial of longer duration.

## Data Availability

Data are available on reasonable request. The individual anonymised data supporting the analyses contained in the manuscript will be made available on reasonable written request from researchers whose proposed use of the data for a specific purpose has been approved. Data will not be provided to requesters with potential or actual conflicts of interest, including individuals requesting access for commercial, competitive or legal purposes. Data access may be precluded for studies in which clinical data were collected subject to legal, contractual or consent provisions that prohibit transfer to third parties. All those receiving access to data will be required to enter into a Data Use Agreement (DUA), which shall contain terms and conditions that are customary for similar agreements and similar companies in the industry. For requests, please email JFP, Kiniksa Pharmaceuticals Chief Medical Officer jpaolini@kiniksa.com.
