# Supplementary material for: Efficacy and safety of mavrilimumab in giant cell arteritis: a phase 2, randomised, double-blind, placebo-controlled trial
Source: Ann Rheum Dis. 2022 Mar 9;81(5):653–61. doi: 10.1136/annrheumdis-2021-221865 (PMC8995812; doi:10.1136/annrheumdis-2021-221865)

Efficacy and safety of mavrilimumab in giant-cell arteritis: a phase 2, randomised, double-blind, placebo-controlled trial

Online Supplemental Material

Maria C. Cid, M.D.,[1]* Sebastian Unizony, M.D.,[2]* Daniel Blockmans, M.D., Ph.D.,[3] Elisabeth Brouwer, M.D., Ph.D.,[4] Lorenzo Dagna, M.D.,[5,6] Bhaskar Dasgupta, M.B.B.S., M.D., F.R.C.P.,[7] Bernhard Hellmich, M.D.,[8] Eamonn Molloy, M.D., M.S., F.R.C.P.I.,[9] Carlo Salvarani, M.D., Ph.D.,[10] Bruce C. Trapnell, M.D.,[11] Kenneth J. Warrington, M.D.,[12] Ian Wicks, M.B., B.S., Ph.D., F.R.A.C.P.,[13] Manoj Samant, Ph.D.,[14] Teresa Zhou, Ph.D.,[14] Lara Pupim, M.D.,[14]
John F. Paolini, M.D., Ph.D.,[14] for the KPL-301-C001 Investigators**
*Drs. Cid and Unizony contributed equally to this article.
**A complete list of the KPL-301-C001 investigators is provided in the Supplementary Appendix.

[1]Vasculitis Research Unit, Department of Autoimmune Diseases, Hospital Clínic, University of Barcelona, Institut d'Investigacions Biomèdiques August Pi i Sunyer (IDIBAPS), Barcelona, Spain; [2]Vasculitis and Glomerulonephritis Center, Division of Rheumatology, Allergy and Immunology, Massachusetts General Hospital, Boston, Massachusetts, United States; [3]General Internal Medicine, UZ Leuven, Leuven, Belgium; [4]Department of Rheumatology and Clinical Immunology, University of Groningen, University Medical Center Groningen, Groningen, The Netherlands; [5]Unit of Immunology, Rheumatology, Allergy and Rare Diseases (UnIRAR), IRCCS San Raffaele Scientific Institute, Milan, Italy; [6]Vita-Salute San Raffaele University, Milan, Italy; [7]Mid and South Essex University Hospitals NHS Foundation Trust, Southend University Hospital, Westcliff-on-sea, United Kingdom; [8]Department of Internal Medicine, Rheumatology and Immunology, Medius Kliniken, Kirchheim unter Teck, Germany; [9]St. Vincent's University Hospital, Dublin, Ireland; [10]Azienda USL-IRCCS di Reggio Emilia and Università di Modena e Reggio Emilia, Reggio Emilia, Italy;

[11]Translational Pulmonary Science Center, Cincinnati Children's Hospital, Cincinnati, Ohio, United States; [12]Mayo Clinic, Rochester, Minnesota, United States; [13]Walter & Eliza Hall Institute, University of Melbourne & Melbourne Health, Melbourne, Australia; [14]Kiniksa Pharmaceuticals Corp., Lexington, Massachusetts, United States.

CONTENTS

**Section**	**Page**
CLINICAL ENDPOINT COMMITTEE	4
INDEPENDENT PULMONARY EVALUATION COMMITTEE	4
METHODS	5
Full Inclusion/Exclusion Criteria	5
Definition of New-Onset and Relapsing/Refractory Giant-Cell Arteritis	6
Study Design	7
Procedures	7
Adjudication of Flare	7
Management of Treatment of Patients Who Experience Flare	8
End Points	8
RESULTS	8
Additional Secondary End Points	8
Adherence to Prednisone Taper in Patients with Sustained Remission	9
Adjudication Concordance	9
Table S1. Patient-Level Relapse Characteristics.	10
Table S2. Proportions of Patients with Elevations in Acute-Phase Reactants at Time of Flare and without Flare	13
Figure S1. Time to First Flare of Giant-Cell Arteritis in Patients with New-Onset Disease and Patients with Relapsing/Refractory Disease	14
Figure S2. Sustained Remission Rate of Giant-Cell Arteritis at Week 26 in Patients with New-Onset Disease and Patients with Relapsing/Refractory Disease	16

CLINICAL ENDPOINT COMMITTEE
Steven Vlad, MD (Chair), Tufts Medical Center
Pascal Hilliquin, MD, Centre Hospitalier Sud Francilien
Paul Monach, MD, VA Boston Healthcare System

INDEPENDENT PULMONARY EVALUATION COMMITTEE
Robert Wise, MD (Chair), The Johns Hopkins University School of Medicine
John S. Ferguson, MD, University of Wisconsin School of Medicine
Violeta Vucinic, MD, University of Belgrade, Medical School
Jonathan Chung, MD, University of Chicago Medicine

METHODS

Full Inclusion/Exclusion Criteria

Inclusion Criteria

- Able and willing to provide written informed consent and to comply with the study protocol
- Age 50 to 85 years inclusive
- Diagnosis of new-onset or relapsing/refractory giant-cell arteritis (see further details below)
- Remission of giant-cell arteritis at or before day 0 (resolution of giant-cell arteritis symptoms and C-reactive protein [CRP] levels less than 1.0 mg/dL or erythrocyte sedimentation rate [ESR] less than 20 mm in first hour), such that the patient can safely participate in the study and follow the protocol-defined procedures, including initiation of the prednisone taper at the protocol-specified starting dose (i.e., ≤60 mg/day)
- At day 0, receiving or able to receive oral prednisone up to 60 mg/day for the treatment of giant-cell arteritis
- If using methotrexate, oral or parenteral up to 25 mg/wk is permitted in screening if started more than 6 weeks before day 0 and should be tapered to zero by day 0
- Willing to receive antiplatelet therapy, depending on investigator’s decision
- Willing to receive treatment for prevention of glucocorticoid-induced osteopenia/osteoporosis, depending on investigator’s decision
- Female patients must be:
    - Postmenopausal, defined as at least 12 months post cessation of menses (without an alternative medical cause), or
    - Permanently sterile following documented hysterectomy, bilateral salpingectomy, bilateral oophorectomy, or tubal ligation or having a male partner with vasectomy as affirmed by the patient, or
    - Nonpregnant, nonlactating, and if sexually active having agreed to use a highly effective method of contraception (i.e., hormonal contraceptives associated with inhibition of ovulation or intrauterine device [IUD], or intrauterine hormone-releasing system [IUS], or sexual abstinence) from the screening visit until the final washout safety follow-up visit 84 ± 3 days from end-of-treatment visit
- Male patients must have documented vasectomy or if sexually active must agree to use a highly effective method of contraception with their partners of childbearing potential (i.e., hormonal contraceptives associated with the inhibition of ovulation or intrauterine device [IUD], or intrauterine hormone-releasing system [IUS], or sexual abstinence) from day 0 until the final washout safety follow-up visit 84 ± 3 days from end-of-treatment visit. Male patients must agree to refrain from donating sperm during the study period.

Exclusion Criteria

- Major surgery within 8 weeks before screening or planned major surgery within 12 months after randomisation
- Transplanted organs (except corneal transplant performed more than 3 months before randomisation)
- Major ischemic event unrelated to giant-cell arteritis within 12 weeks of screening
- Concurrent enrollment in another clinical study, with the exception of observational studies
- Previous treatment with mavrilimumab
- Treatment with any nonbiologic investigational drug therapy within 4 weeks or 5 half-lives of the study agent, whichever was longer, before screening
- Any cell-depleting biologic therapies (e.g., anti-CD20) within 12 months before day 0; or previous treatment with noncell-depleting biologic therapies (e.g., anti–tumor necrosis factor [TNF], anakinra, anti–interleukin 6 [IL-6] receptor [e.g., tocilizumab], or abatacept) within 8 weeks (or 5 half-lives, whichever is longer) before screening
- Treatment with alkylating agents within 12 weeks before screening
- Intramuscular, intra-articular, or intravenous glucocorticoids within 4 weeks before screening
- Receipt of live (attenuated) vaccine within 4 weeks before day 0

- Treatment with hydroxychloroquine, cyclosporine A, azathioprine, cyclophosphamide, or mycophenolate mofetil within 4 weeks before screening
- Female patients who are pregnant, intending to become pregnant, or breastfeeding
- Any condition that, in the opinion of the investigator, could interfere with evaluation of mavrilimumab or interpretation of patient safety or confound the results of the study
- Known history of allergy or reaction to any component of the mavrilimumab or placebo formulation or to any other biologic therapy or prednisone or any of its excipients
- Positive (or 2 indeterminate) QuantiFERON test results
- Clinically significant active infection, including signs/symptoms suggestive of infection, any significant recurrent or chronic infection (including positive hepatitis C virus antibody [HCVAb]), or any episode of infection requiring hospitalization or treatment with intravenous antibiotics within 12 weeks before screening; patients with any opportunistic infection within 6 months before screening
- Patients with chronic active hepatitis B infection as defined below will be excluded from the study:
  - Hepatitis B surface antigen (HbsAg) positive
  - Hepatitis B anti-core antibody positive but anti-surface antibody negative
- Patients at high risk of infection (e.g., history of hereditary or acquired immune deficiency disorder, including history of known human immunodeficiency virus [HIV] infection) or with a history of an infected joint prosthesis at any time with that prosthesis still in situ, leg ulcers, indwelling urinary catheter, or persistent or recurrent chest infections
- History of cancer within the last 10 years, except for basal and squamous cell carcinoma of the skin or in situ carcinoma of the cervix treated and considered cured
- Evidence of clinically uncontrolled respiratory disease. The investigator should review the data from patients' respiratory assessments, including chest x-ray and pulmonary function tests (PFTs), including diffusing capacity for carbon monoxide (DLCO) tests performed during the screening period or within 12 weeks before day 0 if results of prior assessments are available. Available PFT and DLCO assessments must have had values greater than or equal to 60% of predicted for measurements performed and no uncontrolled lung disease. A patient's medical regimen should not have been significantly intensified to control lung disease within 12 weeks before day 0.
- History of chronic respiratory tract infections
- Congestive heart failure, New York Heart Association classification III or IV
- At screening blood tests, any of the following:
  - Aspartate transaminase (AST) >2 × upper limit of normal (ULN)
  - Alanine transaminase (ALT) >2 × ULN
  - Hemoglobin <75 g/L
  - Neutrophils <1.5 × 10^9/L
  - Creatinine clearance (CrCl) <30 mL/min

Definition of New-Onset and Relapsing/Refractory Giant-Cell Arteritis

- New-onset: The new-onset disease cohort includes patients who have been diagnosed within 6 weeks before day 0 using acute-phase reactants, signs/symptoms, and diagnostic criteria
- Relapsing/refractory: (either/or)
  - The relapsing disease cohort includes patients having prior documented diagnosis of giant-cell arteritis as per diagnostic criteria above more than 6 weeks before day 0 and who have active giant-cell arteritis disease defined by acute-phase reactants and signs/symptoms within 6 weeks before day 0
  - The refractory disease patient has had no remission since the diagnosis of disease, as per clinical expectations. Thus, the patient has documentation of prior diagnosis of giant-cell arteritis, as per diagnostic criteria above, more than 6 weeks before day 0; however, presence of acute-phase reactants and signs/symptoms as per above persists within 6 weeks before day 0.

Study Design

The glucocorticoid taper was started at a dose of oral prednisone between 20 mg and 60 mg daily based on prior glucocorticoid dose, disease status, and investigator discretion. The prednisone dose was subsequently tapered over the 26-week double-blind treatment period according to clinical practice and prior clinical trials.[1] Patients starting on 20 mg or 25 mg daily had their doses held at 20 mg or more for the first 3 weeks. Any patient who could not adhere to the protocol-defined steroid taper because of flare/relapse was permitted escape therapy at the discretion of the investigator. During the 12-week washout safety follow-up period, patients could be observed with no additional therapeutic or could be managed with standard of care, which could include corticosteroids, dose modification of corticosteroids, other immunomodulatory agents (with washout period recommended), and/or approved therapies (e.g. tocilizumab), as per the discretion of the investigator.

Study Design reference: 1. Stone JH, Tuckwell K, Dimonaco S, et al. Trial of tocilizumab in giant-cell arteritis. *N Engl J Med*. 2017;**377**(4):317–328.

Procedures

Randomisation, stratified by disease type (new-onset or relapsing/refractory) at baseline, was completed using interactive response technology (Interactive Web Response System). Allocation to treatment groups was done using a central computerized randomisation procedure with a permuted block design and a block size of 5. Patients, investigators, and study personnel were masked to treatment assignments during the study.

Remission was required at the onset of the treatment period to increase the precision in the capture of time to flare (primary outcome) and to streamline the time-to-event statistical analyses.

In addition, the prednisone tapering schedule was designed such that every patient would reach the critical 8-10 mg/day prednisone dose threshold at a similar time frame regardless of the starting dose, thus aligning the time domain for the time-to-event analysis.

Adjudication of Flare

All documented reports of flare were reviewed by an independent, blinded clinical endpoint adjudication committee. Further details are included in the Methods section of the Supplementary Appendix. A contract research organization prepared all documentation of reports of flare that was then reviewed by an independent, blinded clinical endpoint adjudication committee. Flare was defined as a re-increase in acute phase reactants, i.e., CRP level from normal to 1 mg/dL or greater and/or of ESR from less than 20 mm in the first hour to 30 mm or greater, and at least one of the following signs or symptoms attributed by the investigator to new, worsening, or recurrent giant-cell arteritis:

- Cranial symptoms
    - New or recurrent headache or pain or tenderness of the scalp or the temporal artery
    - Visual signs/symptoms, such as ischemic retinopathy, ischemic optic neuropathy, diplopia, amaurosis fugax, etc
    - New or recurrent claudication of the tongue or the masseter muscle, or worsening temporal artery signs and symptoms
    - Transient ischemic attack or stroke related to giant-cell arteritis in the opinion of the investigator
- Extracranial symptoms
    - Classic polymyalgia rheumatica–like symptoms, defined as shoulder and/or hip girdle pain associated with inflammatory morning stiffness
    - New or recurrent claudication in the peripheral circulation (i.e., in one of the extremities)
    - New or worsening angiographic abnormalities detected via MRI, CT/CTA, or PET-CT of the aorta or other great vessels or via ultrasonography of the temporal arteries

Supportive findings included other symptoms that, in the opinion of the investigator, were related to worsening giant-cell arteritis, such as sustained daily recurrent fever with a temperature over 38°C for more than 1 week, chronic anemia, or unexplained weight loss. All elements of the diagnostic work-up pertinent to the investigator diagnosis of a flare/relapse (i.e., the primary efficacy end point) were then reviewed by an independent blinded clinical endpoint committee.

Management of Treatment of Patients Who Experience Flare
Patients who experienced a flare or who could not adhere to the protocol-defined steroid taper because of a flare were treated to ensure their best possible care as follows:

- The patient discontinued the assigned study drug (mavrilimumab or placebo) and was offered escape therapy in accordance with local standard of care, as determined by the investigator, which included dose modifications of glucocorticoids, other immunomodulatory agents, and/or approved therapies (e.g., tocilizumab). The dosages of all concomitant medications used to treat the flare were noted.

Escape glucocorticoid therapy was escalated immediately. Because of possible safety and pharmacologic effects resulting from overlapping exposures to immunomodulatory agents on top of co-administered glucocorticoids, investigators were asked to consider a washout period of at least 35 days (based on elimination half-lives) after the last dose of study drug if deemed clinically appropriate.

End Points

Other secondary end points organized by hierarchy were as follows: time to elevated ESR by week 26, time to elevated CRP by week 26, time to development of giant cell arteritis signs/symptoms or new/worsening vasculitis on imaging by week 26, cumulative prednisone dose at week 26 (including dose received after flare), percent of patients who completed the prednisone taper and had normal ESR by week 26, percent of patients who completed the prednisone taper and had a normal CRP by week 26, percent of patients who completed the prednisone taper and had no new giant cell arteritis signs/symptoms or new/worsening vasculitis on imaging by week 26, and cumulative glucocorticoid dose at the end of the safety follow-up period (week 38). Time to flare by week 26 and sustained remission at week 26 were also assessed in the subgroups of patients with new-onset and relapsing/refractory disease at baseline, although the study was not powered for this assessment, given the limited sample size. Finally, the proportion of patients with elevated ESR or CRP and without giant cell arteritis flare was assessed in a post hoc analysis.

RESULTS

Additional secondary end points
Median (95% CI) time to elevated ESR was significantly longer for mavrilimumab recipients (26·1 [16·1, NE] weeks) compared to placebo recipients (12·1 [8·1, 16·6] weeks; P=0·028) (Table 3). Median (95% CI) time to elevated CRP level was significantly longer for mavrilimumab recipients (NE [8.1, NE]) compared to placebo recipients (12·3 [3·3, 24·1] weeks; P=0·038). Finally, median (95% CI) time to giant-cell arteritis signs and symptoms or new or worsening vasculitis by imaging was NE (NE, NE) for mavrilimumab recipients and was 25·1 (15·1, NE) weeks for placebo recipients (P=0·065). As significance was not reached with this end point, p-values for all following endpoints in the hierarchy are nominal. The mean cumulative prednisone dose by week 26 was 2074 mg in mavrilimumab recipients and 2403 mg in placebo recipients (nominal P=0.067); least-squares mean difference (nominal 95% CI) was –326 mg (–676 mg to 23 mg). A higher proportion of mavrilimumab recipients completed glucocorticoid tapering and had a normal ESR (45·2% and 14·3%, respectively; nominal P=0·020) or no disease signs and symptoms (71·4% vs. 32·1%; nominal P=0·0031) compared with placebo recipients. The proportion of patients completing glucocorticoid taper with a normal CRP level was numerically

higher for mavrilimumab recipients versus placebo recipients (23·8% and 14·3%; nominal P=0·55); not all patients had locally performed CRP measurements.

Adherence to Prednisone Taper in Patients with Sustained Remission

Five patients did not adhere strictly to the prednisone taper but were included in the calculation as having sustained remission. Three patients (two receiving placebo and one receiving mavrilimumab) adhered to the taper but were still receiving prednisone 1 mg daily instead of 0 mg daily at week 26. A fourth patient receiving mavrilimumab adhered to the taper until non-specific temporal headache prompted the investigator to increase the prednisone dose from 1 mg to 5 mg at week 24; ESR and CRP (central) were not elevated at the time of non-specific headache or during the remainder of the 26-week period and the patient did not meet the protocol definition of flare. Finally, a fifth patient receiving placebo adhered to the taper until scalp paresthesia and increased CRP and ESR led the investigator to increase the prednisone dose from 3 mg to 10 mg at week 17; scalp paresthesia was determined as eczema, and the patient assumed the prednisone taper after that point. All other patients with sustained remission adhered to the prednisone taper.

Adjudication Concordance

One placebo-treated patient developed chronic anemia, unexplained weight loss, and elevated ESR at week 24, which was assessed by the investigator as flare but was not confirmed by adjudication because of the absence of definitive symptoms or positive imaging. Active giant-cell arteritis was diagnosed by imaging (PET-CT) in this patient during the safety follow-up at week 30.

Table S1. Patient-Level Relapse Characteristics.

Patient	Treatment	Time of Relapse (week)	ESR at Time of Flare (mm/h)	CRP at Time of Flare (mg/dL)	Prednisone Dose (mg)	GCA Signs and Symptoms at Time of Flare
1	Mavrilimumab	26.1	73	1.8	60	Imaging consistent with worsening vasculitis
2	Mavrilimumab	16.1	33	6.3	4	Cranial: scalp or temporal artery tenderness; extracranial: symptoms of PMR
3	Mavrilimumab	5.7	15	1.5	30	Cranial: N/O localized headache, unexplained m/j pain upon mastication
4	Mavrilimumab	16.1	45	3.2	20	Extracranial: symptoms of PMR
5	Mavrilimumab	15.4	102	7.6	5	Cranial: N/O localized headache, scalp or temporal artery tenderness, unexplained m/j pain upon mastication; extracranial: symptoms of PMR
6	Mavrilimumab	24.3	30	0.1	0	Cranial: N/O localized headache, unexplained m/j pain upon mastication; extracranial: claudication of the extremities, symptoms of PMR; imaging: consistent with worsening vasculitis
7	Mavrilimumab	5	34	1.4	50	Cranial: unexplained m/j pain upon mastication; extracranial: symptoms of PMR
8	Mavrilimumab	16.4	27	1.4	3	Extracranial: symptoms of PMR; imaging: consistent with worsening vasculitis; supportive: unexplained weight loss
9	Placebo	6.4	12	1.5	20	Cranial: unexplained m/j pain upon mastication; extracranial: symptoms of PMR
10	Placebo	17.1	39	2.4	34	Cranial: N/O localized headache, scalp or temporal artery tenderness; extracranial: symptoms of PMR; imaging: consistent with

						worsening vasculitis, evidence of new large vessel vasculitis
11	Placebo	16.3	25	3.4	5	Cranial: N/O localized headache, scalp or temporal artery tenderness; extracranial: symptoms of PMR; imaging: consistent with worsening vasculitis, evidence of new large vessel vasculitis
12	Placebo	16	101	9.0	5	Cranial: N/O localized headache; extracranial: symptoms of PMR;
13	Placebo	20.1	14	1.2	2	Cranial: N/O localized headache, scalp or temporal artery tenderness; extracranial: symptoms of PMR; imaging: consistent with worsening vasculitis; supportive: unexplained weight loss
14	Placebo	12.3	50	2	5	Extracranial: symptoms of PMR;
15	Placebo	25.1	31	1.1	0	Extracranial: symptoms of PMR; imaging: consistent with worsening vasculitis
16	Placebo	11.4	87	2.8	6	Extracranial: symptoms of PMR; imaging: consistent with worsening vasculitis, evidence of new large vessel vasculitis; supportive: unexplained weight loss
17	Placebo	11.7	11	1.6	7	Extracranial: claudication of the extremities; imaging: consistent with worsening vasculitis, evidence of new large vessel vasculitis
18	Placebo	15.1	31	0.8	10	Cranial: N/O localized headache
19	Placebo	10.9	33	1.0	7	Extracranial: symptoms of PMR
20	Placebo	12.3	51	0.6	15	Extracranial: symptoms of PMR
21	Placebo	17	49	1.1	80	Cranial: N/O localized headache, scalp or temporal artery tenderness, unexplained m/j

	pain upon mastication; extracranial: symptoms of PMR

Table S2. Proportions of Patients with Elevations in Acute-Phase Reactants at Time of Flare and without Flare.

Assessment*	Mavrilimumab + 26-wk Prednisone Taper (N=42)	Placebo + 26-wk Prednisone Taper (N=28)	Mavrilimumab + 26-wk Prednisone Taper (N=42)	Placebo + 26-wk Prednisone Taper (N=28)
	Patients with Flare after Remission		Patients without Flare after Remission	
No. of patients (%)	**8 (19·1)**	**13 (46·4)**	**34 (81·0)**	**15 (53·6)**
Presence of elevated C-reactive protein level† or elevated erythrocyte sedimentation rate‡	8 (100·0)	13 (100·0)	20 (58·8)	14 (93·3)
Presence of elevated C-reactive protein level†	7 (87·5)	10 (76·9)	10 (29·4)	11 (73·3)
C-reactive protein level,† mg/dL, median (interquartile range)	1·8 (1·4-6·3)	1·8 (1·2-2·8)	2·6 (1·8-3·0)	2·0 (1·5-3·4)
Presence of elevated erythrocyte sedimentation rate‡	6 (75·0)	9 (69·2)	16 (47·1)	10 (66·7)
Erythrocyte sedimentation rate,‡ mm/hr, median (interquartile range)	40 (33-73)	49 (33-51)	42 (34-62)	54 (42-59)

*Values are no. (%) of patients, except where indicated otherwise.
†Elevated C-reactive protein level (local/central), ≥1 mg/dL.
‡Elevated erythrocyte sedimentation rate, ≥30 mm/hr.

Figure S1. Time to First Flare of Giant-Cell Arteritis in Patients with New-Onset Disease and Patients with Relapsing/Refractory Disease. At baseline, patients had to be in remission (defined as the absence of giant-cell arteritis signs and symptoms and erythrocyte sedimentation rate <20 mm/hr or C-reactive protein level <1 mg/dL) and receiving an oral prednisone dose between 20 mg and 60 mg daily. Patients who discontinued treatment for reasons other than flare were censored for the calculation of time to flare. A) Time to flare in new-onset patients, defined as a diagnosis of giant-cell arteritis within 6 weeks before baseline. The hazard ratio for the comparison of mavrilimumab and placebo was 0.29 with a 95% confidence interval of 0.06 to 1.31. B) Time to flare in relapsing/refractory patients, defined as a diagnosis of giant-cell arteritis more than 6 weeks before baseline. The hazard ratio for the comparison of mavrilimumab and placebo was 0.43 with a 95% confidence interval of 0.14 to 1.30.

A

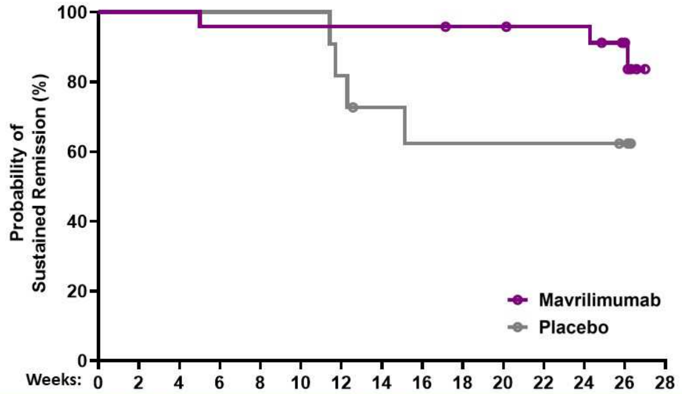

Weeks	0	2	4	6	8	10	12	14	16	18	20	22	24	26	28
Mavrilimumab Patients at Risk, n	24	24	24	23	23	23	23	23	23	22	22	21	21	18	0
Placebo Patients at Risk, n	11	11	11	11	11	11	9	7	6	6	6	6	6	5	0

B

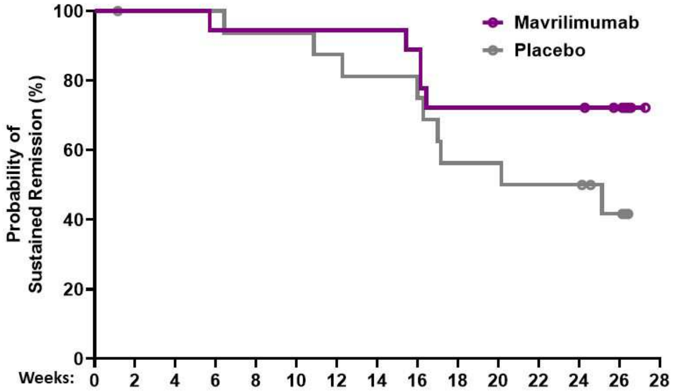

Weeks	0	2	4	6	8	10	12	14	16	18	20	22	24	26	28
Mavrilimumab Patients at Risk, n	18	18	18	17	17	17	17	17	16	13	13	13	13	10	0
Placebo Patients at Risk, n	17	16	16	16	15	15	14	13	13	9	9	8	8	5	0

Figure S2. Sustained Remission Rate of Giant-Cell Arteritis at Week 26 in Patients with New-Onset Disease and Patients with Relapsing/Refractory Disease.

(A) Among the subgroup of patients with new-onset giant cell arteritis at baseline, 91.3% of mavrilimumab recipients and 62.3% of placebo recipients had sustained remission at week 26. **(B)** Among the subgroup of patients with relapsing/refractory disease at baseline, sustained remission at week 26 was observed in 72.2% of mavrilimumab recipients and 41.7% of placebo recipients. The key prespecified secondary efficacy end point of sustained remission was defined as the absence of flare from randomization through week 26. Sustained remission rate was derived by Kaplan-Meier curve analysis.

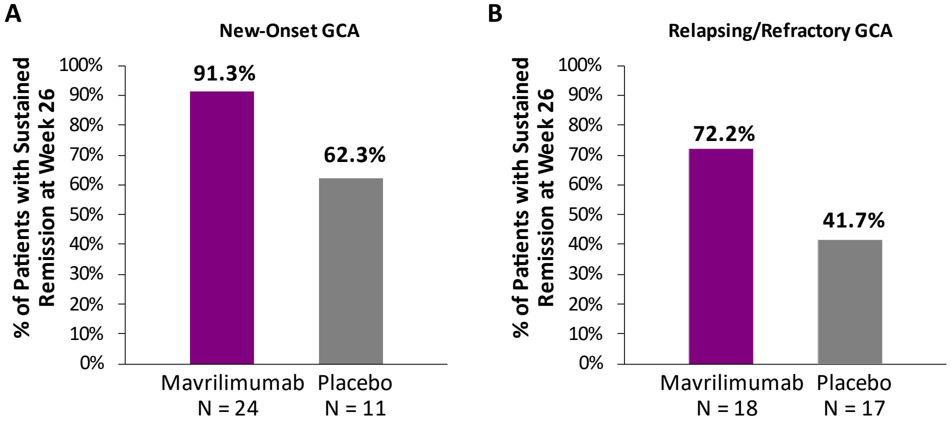

Supplement: Supplementary data [file annrheumdis-2021-221865supp001.pdf]
